# The proline-rich domain of MML-1 is biologically important but not required for localization to target promoters

**DOI:** 10.17912/micropub.biology.000498

**Published:** 2021-11-09

**Authors:** Ainhoa Ceballos, Ruben Esse, Alla Grishok

**Affiliations:** 1 Department of Biochemistry and Molecular Biophysics, College of Physicians and Surgeons, Columbia University, New York, NY 10032, USA; 2 Present affiliation: Oncology Genomics Department, Diagnostica Longwood S.L. 50011 Zaragoza, Spain; 3 Present affiliation: Department of Medical and Molecular Genetics, King’s College London, London SE1 9RT, UK; 4 Present affiliation: Boston University School of Medicine, Department of Biochemistry, Genome Science Institute, Boston, MA 02118, USA

## Abstract

The only representative of the MYC superfamily transcription factors in *C. elegans*, MML-1 (Myc and Mondo-like 1), was shown to promote extended lifespan in a variety of models and to regulate some aspects of *C. elegans* development. This previous research did not involve molecular characterization of MML-1. Here we use available *mml-1* mutant alleles and other reagents to demonstrate that MML-1 is modified by *O*-GlcNAc, binds to promoters of some genes directly regulated by the DOT-1.1 histone methyltransferase complex, and has a role in promoting neuronal migration. Surprisingly, we found that the deletion allele *mml-1(ok849), *which was considered a null, produces an internally truncated protein resulting from an in-frame deletion. Localization of this truncated product to MML-1 target promoters was not impaired. The deleted region of MML-1 is proline-rich, and its function is poorly understood in mammalian homologs of MML-1. Based on our work and previously published data we conclude that the internal proline-rich region of MML-1 is dispensable for DNA binding but is biologically important.

**Figure 1. Deletion of the proline-rich domain of MML-1 does not affect its DNA binding and O-GlcNAc modification:**
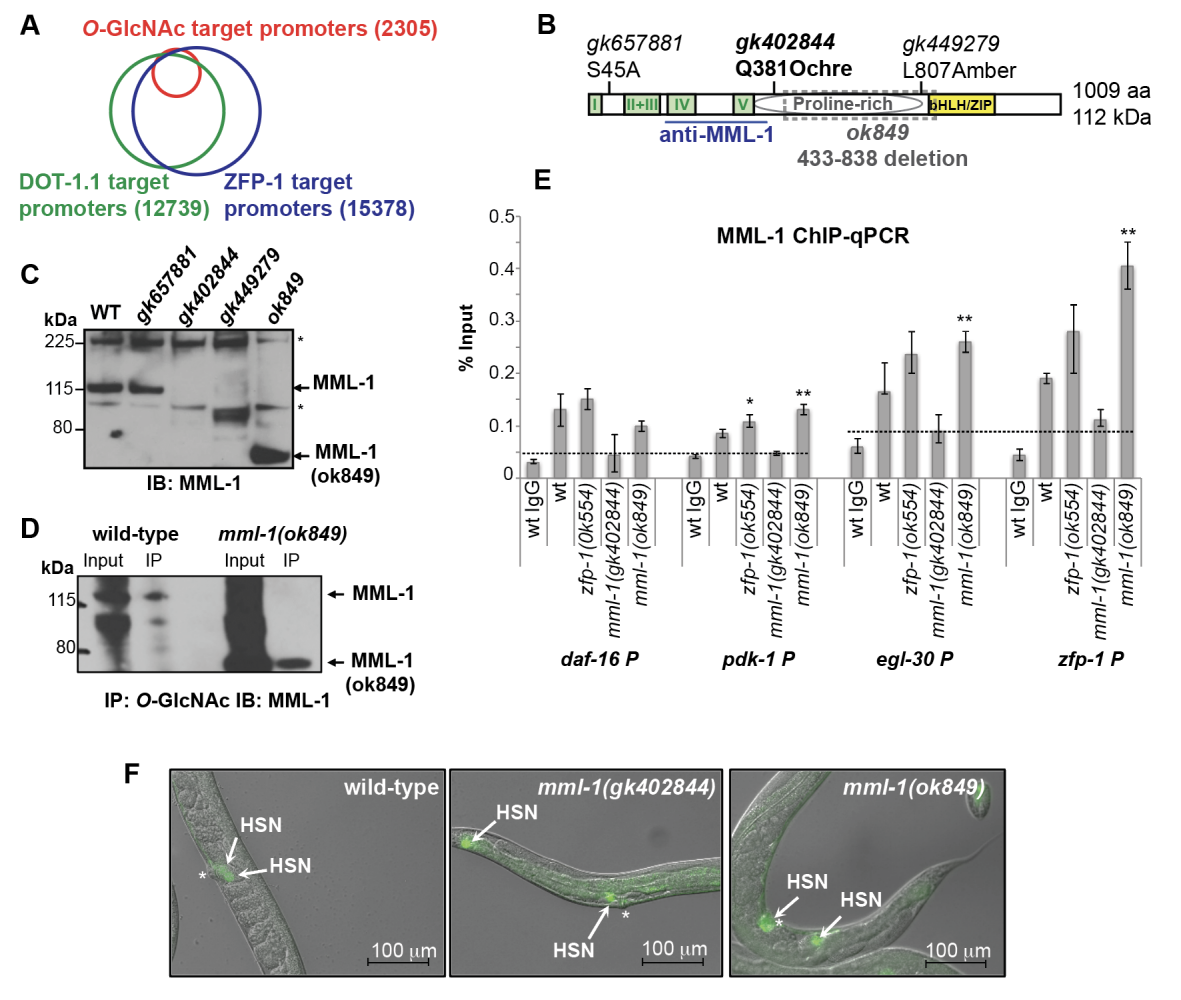
(A)Venn diagram showing an overlap between ZFP-1 and DOT-1.1 chromatin localization peaks (ChIP-chip data, Cecere *et al.* 2013) and *O*-GlcNAc chromatin localization peaks (ChIP-chip data, Love *et al.* 2010). (B) Schematic of the *C. elegans* MML-1 protein: Mondo-specific domains are indicated in green, proline-rich domain is depicted as a grey oval, and the DNA binding/dimerization domain is shown in yellow. The mutant alleles used are indicated above the protein schematic; a loss-of-function *mml-1(ok849)* allele is an in-frame internal deletion, shown by grey box. The region recognized by the anti-MML-1 antibody (Pickett *et al.* 2007) is underlined. (C) Western immunoblotting showing anti-MML-1 antibody specificity using *mml-1* mutant alleles designated on the schematic. Asterisks indicate non-specific bands. (D) Detection of *O*-GlcNAc modification of MML-1: *O*-GlcNAc IP followed by MML-1 western with the N-terminus-specific antibody is shown; the truncated *mml-1(ok849)* product is modified. (E)MML-1 ChIP-qPCR experiment showing MML-1 enrichment at indicated promoters (% Input) in wild type and mutant L3 larvae. Dashed lines denote ChIP background, based on ChIP with unrelated IgG and/or MML-1 ChIP with *mml-1(gk402844)* null mutant extracts. Error bars denote SD of triplicate qPCR runs. Asterisks *, ** and *** denote P values (Student’s t-test) less than 0.05, 0.01 and 0.001, respectively, mutant *vs* wild type or as indicated. MML-1 ChIP-qPCR was repeated three times. (F)Hermaphrodite-specific neuron (HSN) under-migration in *mml-1(gk402844)* and *mml-1(ok849)* mutant worms. HSNs are visualized by the Tryptophan Hydroxylase (TPH) promoter driven GFP reporter marking serotonergic neurons. Asterisks indicate the vulva and arrows denote the HSN position. Images combine Differential Interference Contrast (DIC) and GFP fluorescence; scale bar is 100 micrometers.

## Description

The predominant and ubiquitously expressed *C. elegans* histone H3 lysine 79 methyltransferase DOT-1.1 and its interacting partner, ZFP-1, play important roles in negatively modulating widely and highly expressed genes through promoter binding (Cecere *et al.* 2013) and in creating permissive environment for expression of developmentally regulated genes through enhancers (Esse *et al.* 2019; Esse and Grishok 2020). However, the mechanistic aspects of the ZFP-1/DOT-1.1 complex function are not clear. This study was motivated by the observation that genome-wide promoter-associated ZFP-1 and DOT-1.1 chromatin localization peaks (Mansisidor *et al.* 2011; Cecere *et al.* 2013) overlap with those of the *O*-GlcNAc modification on chromatin observed in wild type *C. elegans* (Love *et al.* 2010) (Figure 1A).Notably, the identities of the *O*-GlcNAc-modified proteins contributing to the *O*-GlcNAc ChIP-chip signals at the select promoters remain unknown. We chose to investigate the *C. elegans* basic helix- loop-helix leucine-zipper (bHLH/ZIP) transcription factor MML-1 (Myc and Mondo-like 1) (Pickett *et al.* 2007; Grove *et al.* 2009) as a candidate *O*-GlcNAc-modified protein, because both c-Myc (Chou *et al.* 1995) and the Mondo B protein (i.e., the Carbohydrate Response Element Binding Protein (ChREBP))(Guinez *et al.* 2011) carry *O*-GlcNAc modification. MML-1 is a 1009 amino acid protein with multiple conserved domains, including the N-terminal Mondo Conserved Regions (I-V) (Pickett *et al.* 2007), which form a glucose sensing module in ChREBP (Abdul-Wahed *et al.* 2017), a proline-rich region, similar to that in ChREBP (Ortega-Prieto and Postic 2019), and a C-terminal DNA-binding and MXL-2 heterodimerization region called bHLH/ZIP (Pickett *et al.* 2007; Grove *et al.* 2009) (Figure 1B).

We obtained several viable *mml-1* mutant alleles generated by the Million Mutation Project (Figure 1B, C) (Thompson *et al*. 2013). These included the potential null mutant *mml-1(gk402844)*, which lacks the MML-1 protein signal recognized by the N-terminus-specific anti-MML-1 antibody (Figure 1C) (Pickett *et al.* 2007) (a generous gift from Dr. Donald Ayer). Notably, the *mml-1(ok849)* deletion allele (Figure 1B), which had been considered null (Johnson *et al.* 2014; Botts *et al.* 2016; Nakamura *et al.* 2016), leads to an in-frame deletion and produces an internally truncated protein (Figure 1C, D). We found that the *O*-GlcNAc-specific antibody RL2 immunoprecipitated both the wild type MML-1 and MML-1(ok849) (Figure 1D), which is consistent with our hypothesis that MML-1 is *O*-GlcNAc-modified. Moreover, both the wild type MML-1 and the MML-1(ok849) binding was detected at the promoters of the previously annotated ChIP targets of ZFP-1 (Mansisidor *et al.* 2011; Cecere *et al.* 2013) (Figure 1E). Notably, *mml-1(gk402844)* showed a background ChIP signal, whereas an increase in ChIP signal compared to that in wild type worms has been observed in *mml-1(ok849)* (Figure 1E). We believe that this increased promoter occupancy by MML-1(ok849) is due to the increased protein stability (Figure 1C, D), which suggests a role of the deleted region in regulating MML-1 dynamics.

Our interest in MML-1 has been driven by the hypothesis that MML-1 and ZFP-1/DOT-1.1 co-regulate common target genes. Since *mml-1(ok849)* phenocopied *mml-1(RNAi)* and behaved like a strong loss-of-function in published functional studies (Johnson *et al.* 2014; Nakamura *et al.* 2016), we were surprised to detect the truncated product by western immunoblotting (Figure 1C). However, the current annotation of the *mml-1(ok849)* allele is correct and predicts an in-frame deletion in MML-1, which results in a 608 aa long MML-1(ok849) (~67 kDa). This predicted size is consistent with our western data (Figure 1C, D).

To gain additional evidence that *mml-1(ok849)* and *mml-1(gk402844)* (a null) shared loss-of-function phenotypes, we analyzed Hermaphrodite-Specific Neuron (HSN) migration. The HSN under-migration phenotype is observed in *zfp-1(ok554)* reduction-of-function mutant, and it is, at least in part, due to the mis-regulation of *pdk-1* expressionin the hypodermis(Kennedy and Grishok 2014). Indeed, both *mml-1(ok849)* and *mml-1(gk402844)* worms displayed defects in HSN migration, 8% (n=49) and 45% (n=49), respectively (Figure 1F), analogous to those that we previously described for *zfp-1(ok554)* (25-30% affected animals) (Kennedy and Grishok 2014).We note that the*mml-1(ok849)* mutant animals exhibited both a less severe and a less penetrant HSN migration phenotype compared to the *mml-1(gk402844)* null. Interestingly,the MML-1/MXL-2 complex has earlier been implicated in cell migration in the male tail, and it acted non-autonomously, in the hypodermis, to promote cell migration (Pickett *et al.* 2007). In sum, these results suggest that MML-1/MXL-2 and ZFP-1/DOT-1.1 may act together in regulating genes expressed in the hypodermis to non-autonomously promote cell migrations during *C. elegans* development.

The MML-1 region deleted in MML-1(ok849) is proline-rich and similar to the proline-rich region in ChREBP, whose function is poorly understood (Ortega-Prieto and Postic 2019). Our findings, together with the previous studies of lifespan control using *mml-1(ok849)* and *mxl-2(tm1516)*, which showed similar phenotypes and behaved like strong loss-of-function mutations (Johnson *et al.* 2014; Nakamura *et al.* 2016), indicate the importance of the proline-rich domain in the biological functions of MML-1. Thus, the existing data indicate that the MML-1(ok849) protein is less active, despite its binding to DNA and its *O*-GlcNAc modification. Moreover, they suggest that a posttranslational activation step targeting the proline-rich region of MML-1, and possibly ChREBP, is required for these DNA-binding proteins to be fully functional in transcription regulation.

## Methods

Western immunoblotting

Approximately 20-25 L3 stage worms were picked directly into the loading buffer and boiled before loading. Proteins were resolved on precast NuPAGE Novex 4–12% Bis-Tris gels (Invitrogen) and transferred to a nitrocellulose membrane (0.45 μm) by semidry transfer (BioRad Trans-Blot SD transfer cell) at a constant current of 0.12A for 1 hour. The blotted membranes were blocked with the blocking buffer (5% non-fat dry milk in TBS-T) at room temperature for 1 hour. Subsequently, they were incubated with the primary antibody overnight at 4°C and with the secondary antibody for 2 hours at room temperature. Three washes with TBS-T buffer were made between and after the incubation with the antibodies. The anti-MML-1 rabbit antiserum raised against the 189-376 aa region was a gift from Dr. Don Ayer, University of Utah. It was used at 1:5000 dilutions.

*O* -GlcNAc immunoprecipitation/ MML-1 western

Synchronized populations of *C. elegans* were grown on agarose 15 cm Petri dishes until larval stage three at 20°C with about 100,000 worms plated per dish. Worms were washed off the plates using isotonic M9 solution and pelleted. The pellet was resuspended in ice-cold RIPA buffer (10 mM Tris-HCl pH 8.0, 0.1% SDS, 1% Triton X-100, 0.1% sodium deoxycholate, 1 mM EDTA, 0.15 M NaCl) supplemented with Halt™ Protease Inhibitor Cocktail (Thermo Fisher Scientific) and sonicated with 30 sec pulses 10 times using Branson microtip sonicator at 10% power in the cold room. The RIPA buffer was used to limit co-immunoprecipitation of proteins not modified by *O*-GlcNAc. Protein extracts were span at 12,000 rpm at 4°C for 10 minutes. Protein concentration was quantified by the Bradford assay, and 8 μg of the anti-*O*-GlcNAc monoclonal antibody (clone RL2, Abcam ab2739) was used with 2.5 mg protein extract in each immunoprecipitation reaction. After 1 hour incubation (4°C) of the immunocomplex, 50μl of Dynabeads™ Protein G suspension (Thermo Fisher Scientific) was added, and the mixture was incubated at 4°C for 1-3 hours on rotation. Beads were recovered with a Dynal® Magnetic Particle Concentrator (Invitrogen). After four washes with the extraction buffer the beads were resuspended in 50μl of gel loading buffer and incubated at 95°C for 5 minutes. Western immunoblotting with the anti-MML-1 rabbit antiserum was performed as described above.

MML-1 ChIP/qPCR

Chromatin immunoprecipitation was performed as described in our earlier publications (Mansisidor *et al.* 2011; Cecere *et al.* 2012; Cecere *et al.* 2013; Gushchanskaia *et al.* 2019). Briefly, 2.5–3mg of the 2% paraformaldehyde cross-linked protein extract from L3 worms was incubated for 1h at 4°C with the specific antibody and the immune complexes were then incubated with 60 μl Dynabeads™ Protein G suspension (Thermo Fisher Scientific) for 1h at 4°C. 1 μl of the anti-MML-1 antiserum was used in each immunoprecipitation reaction. DNA was cleaned up with the Qiagen PCR purification kit. The immunoprecipitated DNA was quantified by qPCR using the ΔΔCt method to calculate the percentage of immunoprecipitation relative to the input.

ChIP/chip data overlap analysis

Genomic regions representing ZFP-1 and DOT-1.1 enrichment were fetched from the modENCODE server (http://www.modencode.org). *O*-GlcNac peaks were obtained from NCBI GEO (GSE18611). For identification of target genes, *C. elegans* transcription unit coordinates were extracted from the UCSC genome browser (RefSeq based on WS220/ce10). A transcription unit was called bound by ZFP-1, DOT-1.1 or *O*-GlcNac if its 1500 bp upstream regions overlapped with a ChIP peak. Overlap analysis was performed in R using the package *valr*(Riemondy *et al.* 2017).

Visualization of *C. elegans* HSN neurons

HSN neurons were detected by using the *zdIs13*[*tph-1::gfp*] transcriptional reporter, as previously described (Kennedy and Grishok 2014).

## Reagents

***C. elegans* strains**

**Table:** 

**Strain Name**	**Genotype**	**Source**
N2	*Wild type Bristol N2*	CGC
RB954	*mml-1(ok849) III*	CGC
RB774	*zfp-1(ok554) III*	CGC
AGK760	*mml-1(gk402844) III.* 4x outcrossed fromVC20787	AGK lab
SK4013	*zdIs13[tph-1p::gfp] IV*	CGC
AGK777	*mml-1(ok849) III; zdIs13[tph-1p::gfp] IV*	AGK lab
AGK778	*mml-1(gk402844) III; zdIs13[tph-1p::gfp] IV*	AGK lab
VC20787	Million Mutations Project strain, contains *mml-1(gk402844) III*	CGC
VC40479	Million Mutations Project strain, contains *mml-1(gk657881) III*	CGC
VC30257	Million Mutations Project strain, contains *mml-1(gk449279) III*	CGC

**Antibodies**

**Table:** 

**Name**	**Source**	**Reference**
anti-*O*-GlcNAc, RL2, mouse monoclonal	Abcam ab2739	(Love *et al.* 2010)
anti-MML-1 antiserum, rabbit, raised against 189-376 aa	Ayer lab (U of Utah)	(Pickett *et al.* 2007)

**Primers used in ChIP/qPCR**

**Table:** 

**Name**	**Sequence**
pdk-1P (F)	AAACAACACATAGACTTGTGCC
pdk-1P (R)	GTACGGTTGTTATCGCTTTCAG
egl-30P3 (F)	CGTGAGTTTGAGTGTCTCTG
egl-30P3 (R)	GATTAGGTTGTGGCTTTCCC
zfp-1P (F)	GTCGTCAATTCTATTTCTCGT
zfp-1P (R)	GATAGTAGCCGAAAGGAACAG
daf-16P (F)	GATTCTCCCTCTCCGTTCAC
daf-16P (R)	GTGATGAAGAAGGTGGTCTC
